# Luteolin attenuates inflammation and apoptosis in LPS-induced ALI mice by activating the HGF/c-Met pathway

**DOI:** 10.3389/fphar.2025.1641486

**Published:** 2025-07-29

**Authors:** Jihong Bi, Yan Li, Lin Lu, Tingting Yin, Shaohua Mao, Run Zhang, Juan He, Xing Ni, Kai Wang, Jirui Bi

**Affiliations:** ^1^ Neurocritical Care Group, Department of Encephalopathy, The Second Affiliated Hospital of Anhui University of Chinese Medicine, Hefei, China; ^2^ Neurocritical Care Group, Department of Neurology, The First Affiliated Hospital of Anhui Medical University, Hefei, China; ^3^ Department of Respiratory, The Second Affiliated Hospital of Anhui University of Chinese Medicine, Hefei, China; ^4^ Neurocritical Care Group, Department of Nuerology, The Third Affiliated Hospital of Anhui Medical University, Hefei, China

**Keywords:** luteolin, hepatocyte growth factor, acute lung injury, acute respiratory distress syndrome, inflammation, apoptosis

## Abstract

**Objective:**

To investigate the protective effects of luteolin on ALI induced by lipopolysaccharide (LPS) in male mice and to explore the underlying mechanisms.

**Methods:**

A two-sample Mendelian randomization (MR) analysis was conducted to assess the causal relationship between hepatocyte growth factor (HGF) and acute respiratory distress syndrome (ARDS). Network pharmacology analysis was employed to identify herbs that mitigate inflammation and apoptosis by upregulating HGF expression and to determine the hub active ingredients. These findings were subsequently validated through *in vivo* experiments using an LPS-induced ALI mouse model. Lung histopathological examination, enzyme-linked immunosorbent assay, and Western blot analysis were performed to evaluate relevant biomarkers of inflammation and apoptosis.

**Results:**

The MR analysis demonstrated a causal effect of HGF on ARDS (IVW: β = −1.120, OR = 0.326, 95% CI = 0.116–0.916, P = 0.033). Among herbs associated with upregulating HGF expression, *Salvia miltiorrhiza Bunge* is involved in the negative regulation of apoptotic process and positive regulation of cell population proliferation. Luteolin was identified as the hub active ingredient extracted from *S. miltiorrhiza Bunge*. In LPS-induced ALI mice, luteolin significantly alleviated histopatholocial damage, inflammation, and apoptosis in the lungs. However, these protective effects were abrogated by c-Met inhibition.

**Conclusion:**

Luteolin attenuates inflammation and apoptosis in the lungs of LPS-induced ALI mice via activation of the HGF/c-Met pathway.

## 1 Introduction

Acute lung injury (ALI) is a severe clinical syndrome characterized by diffuse alveolar injury, hypoxemia, and respiratory distress (Liu et al., 2022). ALI can rapidly progress to acute respiratory distress syndrome (ARDS), leading to severe irreversible tissue damage (Matthay et al., 2024; Bos and Ware, 2022). ARDS is observed in 10.4% of patients in the intensive care unit, with a mortality rate as high as 40.0% (Bellani et al., 2016). Despite significant advancements in the treatment of ALI/ARDS, effective therapeutic strategies to reduce mortality or improve the prognosis of patients remain limited (Bellani et al., 2016; Meyer et al., 2021; Ning et al., 2023). Therefore, it is of great importance to explore novel therapeutic targets and drugs for the treatment of ALI/ARDS.

The inflammation and apoptosis are critically involved in the pathogenesis and progression of ALI/ARDS (Kim et al., 2025; Wang Y. et al., 2023; Han et al., 2025). Activation of inflammatory cells results in the secretion of inflammatory cytokines, such as interleukin (IL)-1β, IL-6, and tumor necrosis factor (TNF)-α, which promote injury to alveolar epithelial cells and vascular endothelial cells (Wang et al., 2022; Wang et al., 2024). Hepatocyte growth factor (HGF) regulates neurogenesis, organogenesis, and tissue remodeling during epithelial wound healing, tissue regeneration, and cancer invasion (Chaparro et al., 2015). HGF binds to and activates the high-affinity cell-surface receptor c-Met, thereby initiating a wide range of biological activities (Molnarfi et al., 2015). For instance, it promotes cell survival through activation of the PI3K/Akt pathway (Xiao et al., 2001). Previous studies have shown that HGF secreted by mesenchymal stem cells (MSCs) exerts endothelial barrier-protective effects *in vitro* and mitigates lung injury *in vivo* (Meng et al., 2019; Yang et al., 2015). These anti-apoptotic effects of HGF on epithelial and endothelial cells are mediated by activation of the HGF/c-Met/PI3K/Akt pathway (Nakagami et al., 2001; Ito et al., 2014). Additionally, increased HGF levels following HGF gene transfection into the lungs of mice with bleomycin-induced injury have been observed to decrease TNF-α and IL-6 levels (Watanabe et al., 2005). Another study also revealed that HGF attenuates allergic airway inflammation in a murine model (Ito et al., 2005). These findings indicate that modulating HGF levels may be an effective strategy to alleviate inflammation and apoptosis, which is crucial for the management of ALI/ARDS.

The natural herbs have demonstrated beneficial effects in mitigating lung damage. *Lonicerae japonicae flos*, *Scutellaria baicalensis*, *Bupleurum*, and emodin have been shown to attenuate lung injury induced by human respiratory syncytial virus and silica by inhibiting inflammation and apoptosis (An et al., 2023; Liu et al., 2021). Moreover, several herbs have been demonstrated to upregulate the expression of HGF. Sho-saiko-to extract facilitates liver regeneration in partially hepatectomized rats by elevating HGF levels in the liver and spleen (Ono et al., 2000). *Resina Draconis* ameliorates acute hepatic injury and promotes liver cell proliferation following partial hepatectomy through the HGF pathway (He et al., 2020). However, the complex composition of herbal medicines poses a challenge in distinguishing between active and toxic constituents (Ma et al., 2023). Therefore, identifying the therapeutic effects of active ingredients extracted from herbs on ALI/ARDS is essential for improving patient outcomes. Nevertheless, the investigation of active ingredients targeting HGF that exert protective effects on ALI/ARDS by inhibiting inflammation and apoptosis has been rarely reported.

Luteolin (3,4,5,7-tetrahydroxy flavone), a bioactive flavonoid, is widely distributed in various fruits and vegetables, as well as in medicinal plants such as *Lonicera japonica*, *Codariocalyx motorius*, and *Salvia miltiorrhiza Bunge* (Muruganathan et al., 2022; Lv et al., 2025; Aziz et al., 2018). Previous studies have demonstrated that the peak plasma concentration of luteolin is achieved within 1–2 h following oral administration and remains in the circulation for several hours (Chen Ting et al., 2007; Lu et al., 2011; Li et al., 2013; Kure et al., 2016). Luteolin exhibits relatively high bioavailability and a sufficiently slow metabolic rate, enabling its metabolites to exert biological activities in *in vivo* models (Ali and Siddique, 2019). Extensive research has demonstrated that luteolin possesses numerous beneficial properties, including anti-inflammatory, antioxidant, anti-tumor, and anti-apoptotic effects (Lv et al., 2025; Aziz et al., 2018; Duggal et al., 2015). Chen et al. showed that luteolin suppresses inflammatory responses induced by lipopolysaccharide (LPS) in mouse alveolar macrophages by inhibiting the production of reactive oxygen species and pro-inflammatory cytokines (Chen C-Y. et al., 2007), suggesting that luteolin may have potential therapeutic applications for the treatment of inflammatory lung disorders. An increasing number of studies utilizing animal models have demonstrated that luteolin exhibits therapeutic effects on multiple pulmonary diseases, such as ALI, chronic obstructive pulmonary disease, asthma, and pulmonary fibrosis (Das et al., 2003; Li et al., 2023; Ren et al., 2023; Cao et al., 2025). However, whether luteolin exerts its beneficial effects in LPS-induced ALI by targeting HGF remains to be elucidated.

In this study, by integrating Mendelian randomization (MR) analysis, network pharmacology analysis, and *in vivo* experiments, we found that luteolin alleviates LPS-induced ALI in mice by inhibiting apoptosis and inflammation through the HGF/c-Met pathway.

## 2 Materials and methods

### 2.1 Study design

**FIGURE 1:**
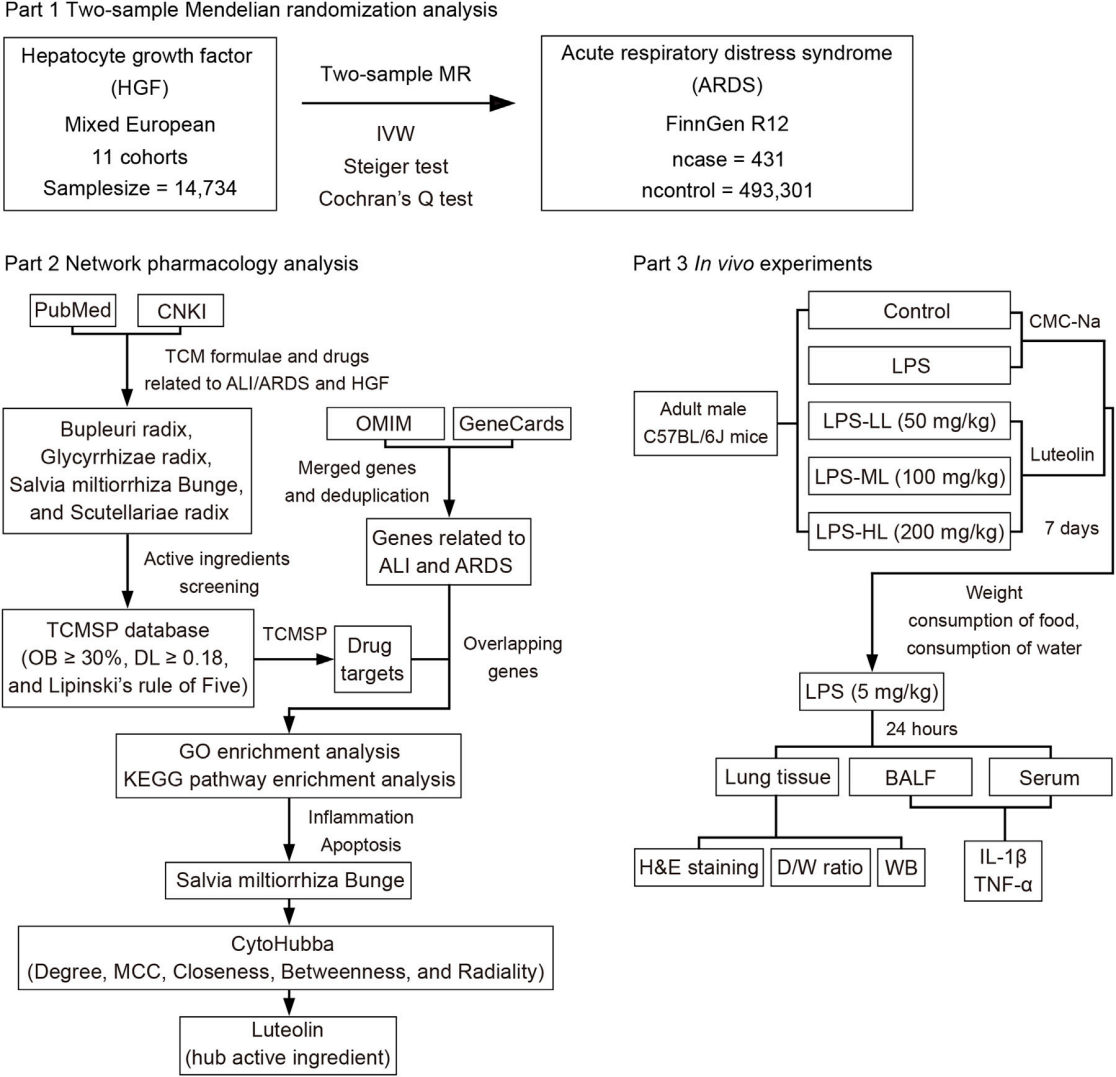
Diagram of three primary parts in the study design.

The current study primarily consists of three components, as depicted in the flowchart shown in Figure 1. Initially, a two-sample MR analysis was utilized to evaluate the causal effects of HGF on ARDS. Then, network pharmacology analysis was conducted to identify the natural herbs associated with HGF and ALI/ARDS, and to select the hub active ingredients. Finally, *in vivo* experiments were designed to validate the protective effects of the hub active ingredient on LPS-induced ALI in mice.

### 2.2 Two-sample MR analysis

#### 2.2.1 Data sources

The datasets for MR analysis were sourced from publicly available genome-wide association study (GWAS) datasets based on the European population. The summary dataset of HGF was acquired from a genome-wide protein quantitative trait locus study of 91 circulating inflammatory proteins in 14,824 participants from 11 cohorts (https://www.ebi.ac.uk/gwas) (Zhao et al., 2023). The GWAS summary dataset of ARDS was obtained from the FinnGen Consortium R12 (https://www.finngen.fi/en), which included 431 patients with ARDS and 493,301 control individuals (Kurki et al., 2023). Detailed information regarding the datasets is presented in the original studies and Supplementary Table S1. Ethics approval was not required for our study, as the ethics approval and informed consent of the data used in this study have been obtained in the original research.

#### 2.2.2 Instrumental variables selection

To estimate causal effects using MR analysis, three basic assumptions must be met to ensure the validity of instrumental variables (IVs) (Emdin et al., 2017): (1) IVs are strongly associated with ARDS; (2) IVs are not associated with any confounders; (3) IVs affect ARDS exclusively through HGF. Specifically, the high-quality IVs included in this study were screened through the following procedures. First, single-nucleotide polymorphisms (SNPs) with a genome-wide significance threshold of p-value < 5e-8 were selected. Second, SNPs with high linkage disequilibrium (r^2^ = 0.001 and window size = 10,000 kb) were removed by filtering against the 1,000 Genomes European reference panel. Then, F-statistics were calculated as an indicator of the strength of SNPs, and those with F-statistic <10 were excluded to avoid weak instrument bias in MR analysis (Liu et al., 2024). Finally, outliers among SNPs were detected using the MR-PRESSO global test, and outliers were removed until the global test yielded a p-value >0.05 (Verbanck et al., 2018). The remaining SNPs were utilized for conducting MR analysis.

#### 2.2.3 MR analysis and sensitivity analysis

In our study, only two SNPs were selected after a series of stringent screening steps. The inverse variance weighted (IVW) method was employed to assess the causal estimates of HGF on ARDS. The IVW method estimates the causal effect by combining the Wald ratio estimates of each SNP (Burgess et al., 2013). It can provide an unbiased causal estimate and sufficient statistical power when there is an absence of horizontal pleiotropy among the IVs (Hemani et al., 2018). Sensitivity analysis was conducted to evaluate the robustness of the causal estimates. Cochran’s Q test was used to quantify the heterogeneity among the IVs. No heterogeneity was observed as the p-value >0.05 (Burgess et al., 2017). Due to the insufficient number of SNPs (fewer than three), horizontal pleiotropy analysis was not performed using MR-Egger regression intercept analysis and leave-one-out analysis (Bowden et al., 2015). Additionally, the Steiger test was conducted to infer the causal direction between the exposure and outcome (Hemani et al., 2017). The results of MR analysis were reported in accordance with the STROBE-MR guideline (Skrivankova et al., 2021).

### 2.3 Network pharmacology analysis

#### 2.3.1 Screening of natural herbs

To identify potential herbs with protective effects against ALI/ARDS by upregulating the expression of HGF, we retrieved literature in Medline (PubMed, https://pubmed.ncbi.nlm.nih.gov) and China National Knowledge Infrastructure (CNKI, http://www.cnki.net) to summarize relevant traditional Chinese medicine (TCM) formulae and single herbs associated with ALI/ARDS and HGF. The search terms included the following keywords: “traditional Chinese medicine,” “Chinese herbal medicine,” “natural product,” “acute lung injury,” “acute respiratory distress syndrome,” and “hepatocyte growth factor” (Ma et al., 2023). The herbs and formulae were listed, and commonly shared herbs were obtained. Ultimately, four herbs were selected for network pharmacology analysis: *Bupleuri radix*, *Glycyrrhizae radix*, *S. miltiorrhiza Bunge*, and *Scutellariae radix*. Detailed information on the TCM formulae is provided in Supplementary Table S2.

#### 2.3.2 Screening of active ingredients

The active ingredients of the four selected herbs were screened based on the following criteria in the Traditional Chinese Medicine Systems Pharmacology Database and Analysis Platform (TCMSP) database (https://www.91tcmsp.com) (Lipinski et al., 2001; Liu et al., 2025): oral bioavailability (OB) ≥ 30%, drug-likeness (DL) ≥ 0.18, and compliance with Lipinski’s rule of Five. The SMILES structures of each active ingredient were retrieved from the PubChem database (https://pubchem.ncbi.nlm.nih.gov). Subsequently, the SMILES data were entered into the SwissADME database (http://www.swissadme.ch) for screening based on pharmacokinetics properties, including high gastrointestinal absorption and drug-likeness without violations. The active ingredients that met these criteria were selected for further target acquisition.

#### 2.3.3 Shared targets of herbs and disease

Potential targets for each active ingredient of the four selected herbs were obtained from the TCMSP. Genes associated with ALI and ARDS were retrieved from the Online Mendelian Inheritance in Man (OMIM, https://omim.org) and GeneCards (https://www.genecards.org) with a minimum score of ≥10.00 (Liu et al., 2025). All identified targets and genes were imported into the Uniprot database (https://www.uniprot.org) to obtain the corresponding UniProt IDs. First, the genes of each disease phenotype obtained from the two databases were merged and deduplicated. Then, the overlapping genes between ALI and ARDS were identified. Finally, the shared genes between each herb and ALI/ARDS were determined using Venn diagram tools (http://bioinformatics.psb.ugent.be/webtools/Venn) and were used for further analysis.

#### 2.3.4 GO and KEGG enrichment analysis

Gene Ontology (GO) enrichment analysis was conducted to determine the cellular component (CC), molecular function (MF), and biological process (BP) items based on massive genetic information. The Kyoto Encyclopedia of Genes and Genomes (KEGG) pathway enrichment analysis is a systematic approach to analyze gene functions within the context of gene and molecular networks. GO and KEGG enrichment analyses were performed to identify the pathways involved in the shared genes between the herbs and ALI/ARDS using the DAVID tools (https://davidbioinformatics.nih.gov). The top 10 significantly enriched GO terms and KEGG pathways with a false discovery rate (FDR) threshold of <0.05 were selected. Consequently, the herb exerting protective effects on ALI/ARDS by regulating the apoptotic process and inflammatory pathways was chosen for further exploration of its hub active ingredients. Heatmap was plotted by an online platform for data analysis and visualization (https://www.bioinformatics.com.cn) (Tang et al., 2023).

#### 2.3.5 Hub active ingredients selection

Cytoscape software (version 3.10.1) was employed to visualize the network of herb-active ingredient-shared gene. The cytoHubba plugin (algorithms of Degree, MCC, Closeness, Betweenness, and Radiality) was used to perform cluster analysis of the network to identify the hub active ingredients (Chin et al., 2014). The network diagram was constructed, and the node colors were adjusted according to the degree values.

### 2.4 *In vivo* experiments

#### 2.4.1 Reagents

Lipopolysaccharide (LPS, *Escherichia coli* O55:B5, Cat: L8880) was purchased from Solarbio Science & Technology, China. Luteolin (Cat: T1027), SU11274 (Cat: T6154), and tribromoethyl alcohol (Cat: T0807) were purchased from Topscience, USA. Sodium carboxymethyl cellulose (CMC-Na, 800–1200mpa.s, USP class, Cat: C104985) was purchased from Aladdin, China. Primary and secondary antibodies, including rabbit-anti cleaved-Caspase 3 (Cat: AF7022), rabbit-anti Bax (Cat: AF0120), rabbit-anti Bcl-2 (Cat: AF6139), rabbit-anti HGF (Cat: DF6326), rabbit-anti PI3K (Cat: AF6241), rabbit-anti p-PI3K (Cat: AF3241), rabbit-anti AKT (Cat: AF6261), rabbit-anti p-AKT (Cat: AF0016), rabbit-anti GAPDH (Cat: AF7021), and goat anti-rabbit IgG (H + L) HRP (Cat: S0001) were purchased from Affinity Biosciences, China. Enhanced BCA protein assay kit (Cat: P0010), one step TdT-mediated dUTP Nick-End Labeling (TUNEL) apoptosis assay kit (Cat: C1089), and Western blot (WB) blocking buffer (Cat: P0023B) were purchased from Beyotime Biotechnology, China. Enzyme-linked immunosorbent assay (ELISA) kits for IL-1β (Cat: MK2776B) and TNF-α (Cat: MK2868B) were purchased from Jiangsu Sumeike Biological Technology, China. Enhanced chemiluminescence kit (Cat:32109) was purchased from Thermo Scientific, United States.

#### 2.4.2 Animals

Adult male C57BL/6J mice, aged 8–10 weeks and weighing 22–24 g, were procured from Ziyuan Experimental Animal Science and Technology Company (SCXK[Zhe] 2019-0004, Hangzhou, China). The mice were housed in a specific pathogen-free environment maintained at 21°C–25°C with a 12-h light-dark cycle. The animals were allowed to access food and water freely. Throughout the study, efforts were made to minimize the suffering of the mice and to reduce the number of animals used. This study complied with national legislation regarding the Care and Use of Animals. All experimental protocols were approved by the Laboratory Animal Ethics Committee of Anhui University of Chinese Medicine (ID: AHUCM-mouse-2024212). All animals were acclimatized to the laboratory conditions for 7 days before the experiments.

#### 2.4.3 LPS-induced ALI

In the animal studies, the doses of luteolin used ranged from 10 to 100 mg/kg per day (Cao et al., 2025; Vajdi et al., 2023). In the present study, the mice were randomly assigned to five groups (n = 6 per group): Control, LPS, LPS + low dose luteolin (LPS-LL, 50 mg/kg), LPS + medium dose luteolin (LPS-ML, 100 mg/kg), and LPS + high dose luteolin (LPS-HL, 200 mg/kg). In the treatment groups, luteolin (dispersed in 0.5% CMC-Na) was administered by intragastric gavage once daily for seven consecutive days. The Control and LPS groups received an equal volume of 0.5% CMC-Na. On the seventh day, 2 hours after the last intragastric administration, LPS (5 mg/kg) was administered intraperitoneally to the mice in the LPS and three luteolin-treated groups. Saline was administered instead of LPS in the Control group. To investigate whether luteolin exerted protective effects on ALI through activation of c-Met, SU11274 (0.18 mg/kg) was administered intraperitoneally 30 min before luteolin administration each day (Miao et al., 2020). After 24 h, the mice were anesthetized with an intraperitoneal injection of 1.25% tribromoethyl alcohol. Bronchoalveolar lavage fluid (BALF), serum, and lung tissues were collected and stored at −80 °C for further analysis. For hematoxylin and eosin (H&E) staining, the tissues were washed with cold phosphate buffered saline (PBS) and fixed in 4% paraformaldehyde solution.

#### 2.4.4 Weight and consumption of food and water

To evaluate the potential toxicity of intragastrically administered luteolin at low, medium, and high doses in mice, the weight and food and water consumption were monitored over a 7-day period during drug administration.

#### 2.4.5 Lung dry/wet weight ratio

The dry/wet weight ratio (D/W ratio) was calculated to assess the extent of lung tissue edema. Briefly, the middle lobe of the right lung was harvested and weighed to obtain the wet weight. The lung was then dried in an oven at 80°C for 48 h, and the dry weight was determined. The D/W ratio was calculated using the formula: (dry weight/wet weight) × 100%.

#### 2.4.6 Histopathologic assay

A standard H&E staining procedure was performed to evaluate histological changes in the lung. Briefly, lung tissues were embedded in paraffin and sectioned into 4-µm slices (Leica, CM3050S, Germany). After deparaffinization and ethanol-based rehydration, the sections were stained with hematoxylin and eosin. Pathological images were observed and captured using a microscope with light mode (Leica, DM6 B, Germany). Subsequently, lung injuries were evaluated and scored at a magnification of ×200, as previously described (Kong et al., 2021; Matute-Bello et al., 2001). Each section was divided into five equal segments, and the mean score was calculated. The scoring was independently performed by two investigators (J.H. Bi and Y. Li) in accordance with standard protocols. Any discrepancies were resolved by a third investigator (J.R. Bi).

#### 2.4.7 TUNEL staining

Following deparaffinization and ethanol-based rehydration, TUNEL staining was performed to assess apoptosis in lung tissue using a TUNEL assay kit, according to the manufacturer’s instructions. For each mouse, three sections were selected, and the number of TUNEL-positive cells was quantified. Five random fields were chosen from each section, and the total number of TUNEL-positive cells in each field was counted within a predefined rectangular area. The total number of apoptotic cells was determined by summing the counts from the 15 fields for each mouse.

#### 2.4.8 Enzyme-linked immunosorbent assay

BALF and serum samples were centrifuged at 3000 rpm for 15 min at 4°C, and the supernatants were collected. The levels of IL-1β and TNF-α were measured using ELISA kits according to the instructions provided by the manufacturer.

#### 2.4.9 Western blot

Total protein lysates were prepared from lung tissues in lysis buffer (150 mM NaCl, 50 mM Tris-HCl 264 (pH 7.4), 1 mM EDTA, 1 mM PMSF, 1% sodium deoxy- 265 cholate, 1% Triton X-100, and 0.1% SDS). The lysates were centrifuged at 4°C, 12,000 rpm for 10 min, and then boiled for 15 min. The total protein concentration was determined using a Bicinchoninic Acid Assay. Samples were electrophoresed on SDS-polyacrylamide gels and then transferred to polyvinylidene difluoride membranes (Millipore, United States, Cat: IPVH00010). After blocking in blocking buffer for 2 h at room temperature, the membranes were incubated at 4°C overnight with primary antibodies: rabbit-anti cleaved-Caspase 3 (1:1,000), rabbit-anti Bax (1:1,000), rabbit-anti Bcl-2 (1:1,000), rabbit-anti HGF (1:1,000), rabbit-anti PI3K (1:1,000), rabbit-anti p-PI3K (1:1,000), rabbit-anti AKT (1:1,000), rabbit-anti p-AKT (1:1,000), and rabbit-anti GAPDH (1:1,000). After three washes with PBST, the membranes were incubated with secondary antibody: goat anti-rabbit IgG (H + L) HRP (1:10000) for 2 h at room temperature. The membrane signals were detected using an enhanced chemiluminescence kit, and the images were captured using a Tanon developer (Tanon Fine-do X6, China).

### 2.5 Statistical analysis

In this study, MR analysis was conducted using R version 4.3.2 within R Studio (R Foundation for Statistical Computing, Vienna, Austria) with the TwoSampleMR package (version 0.5.7) (Yavorska and Burgess, 2017). The causal estimates were presented as odds ratios (ORs) with 95% confidence intervals (95% CI). For *in vivo* experiments, all data with normal distribution were presented as mean ± SD. One-way analysis of variance (ANOVA) followed by Bonferroni post-hoc test was used to analyze differences among groups. Repeated measures two-way ANOVA followed by Bonferroni post-hoc test was used to analyze changes in weight, and food and water consumption among groups. Statistical analysis was performed using SPSS software (version 22.0; IBM Inc., United States). ImageJ (version 1.54f, United States) was used to analyze the band intensities in WB results. All graphs were generated using GraphPad Prism (version 8.4.2, United States). Figures were processed using Adobe Photoshop CS6 (Adobe, United States). A p-value <0.05 was considered as statistically significant.

## 3 Results

### 3.1 Causal effect of HGF on ARDS

**FIGURE 2:**
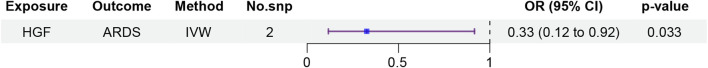
Causal effects of hepatocyte growth factor (HGF) on acute respiratory distress syndrome (ARDS).

Following a series of screening procedures, two SNPs associated with HGF were selected as IVs (Supplementary Table S3). The F-statistics for each SNP were greater than 10, indicating the absence of weak IVs. The results obtained from MR analysis revealed a causal effect of HGF on ARDS (IVW: β = −1.120, OR = 0.326, 95% CI = 0.116–0.916, P = 0.033). This suggests that a genetically determined increase of 1 standard deviation HGF levels is associated with a 67.4% reduction in the risk of ARDS (Figure 2). The findings from the Steiger test further supported that HGF is more likely to be the causal factor for ARDS. No evidence of heterogeneity was detected in the Cochran’s Q test (Supplementary Table S4).

### 3.2 Selection of herbs related to HGF and ALI/ARDS

Numerous TCM formulae have been validated to exhibit protective effects against ALI, such as Qing Fei Pai Du decoction, Qingyi decoction, Ge Gen Qin Lian decoction, and Xuebijing (Zhang C. et al., 2023; Wang Zhengjian et al., 2023; Ding et al., 2020; Ye et al., 2023; Zhang Ganchun et al., 2023). Studies focusing on the effects of TCM formulae on the upregulation of HGF expression have been verified by Yi Shen Huo Xue decoction and Sho-saiko-to extract (Ono et al., 2000; Wu et al., 2015; Feng et al., 2023). The overlapping components present in the formulae that have protective effects on ALI and can upregulate the expression of HGF were retained, including *Bupleuri radix*, *G. radix*, *S. miltiorrhiza Bunge*, and *Scutellariae radix* (Supplementary Table S2).

### 3.3 Active ingredients and shared genes screening

In accordance with the screening criteria, 80, 51, 26, and 8 active ingredients were identified in *G. radix*, *S. miltiorrhiza Bunge*, *Scutellariae radix*, and *Bupleuri radix*, respectively (Supplementary Table S5). These ingredients were utilized for target identification in the TCMSP. After removing duplicates, we ultimately obtained 221, 132, 98, and 172 targets in *G. radix*, *S. miltiorrhiza Bunge*, *Scutellariae radix*, and *Bupleuri radix*, respectively (Supplementary Table S6). The genes associated with ALI and ARDS were retrieved from the GeneCards and OMIM databases. After removing duplicates, 1,587 and 2,130 genes were identified for ALI and ARDS, respectively (Supplementary Table S6). Among these genes, 819 overlapping genes were identified for ALI and ARDS (Supplementary Figure S1A). Then, the shared genes of the four herbs and ALI/ARDS were determined using Venn diagram tools. Consequently, 87, 48, 32, and 74 shared genes were identified between ALI/ARDS and *G. radix*, *S. miltiorrhiza Bunge*, *Scutellariae radix*, and *Bupleuri radix*, respectively (Supplementary Table S7; Supplementary Figure S1A).

### 3.4 GO and KEGG enrichment analysis

**FIGURE 3:**
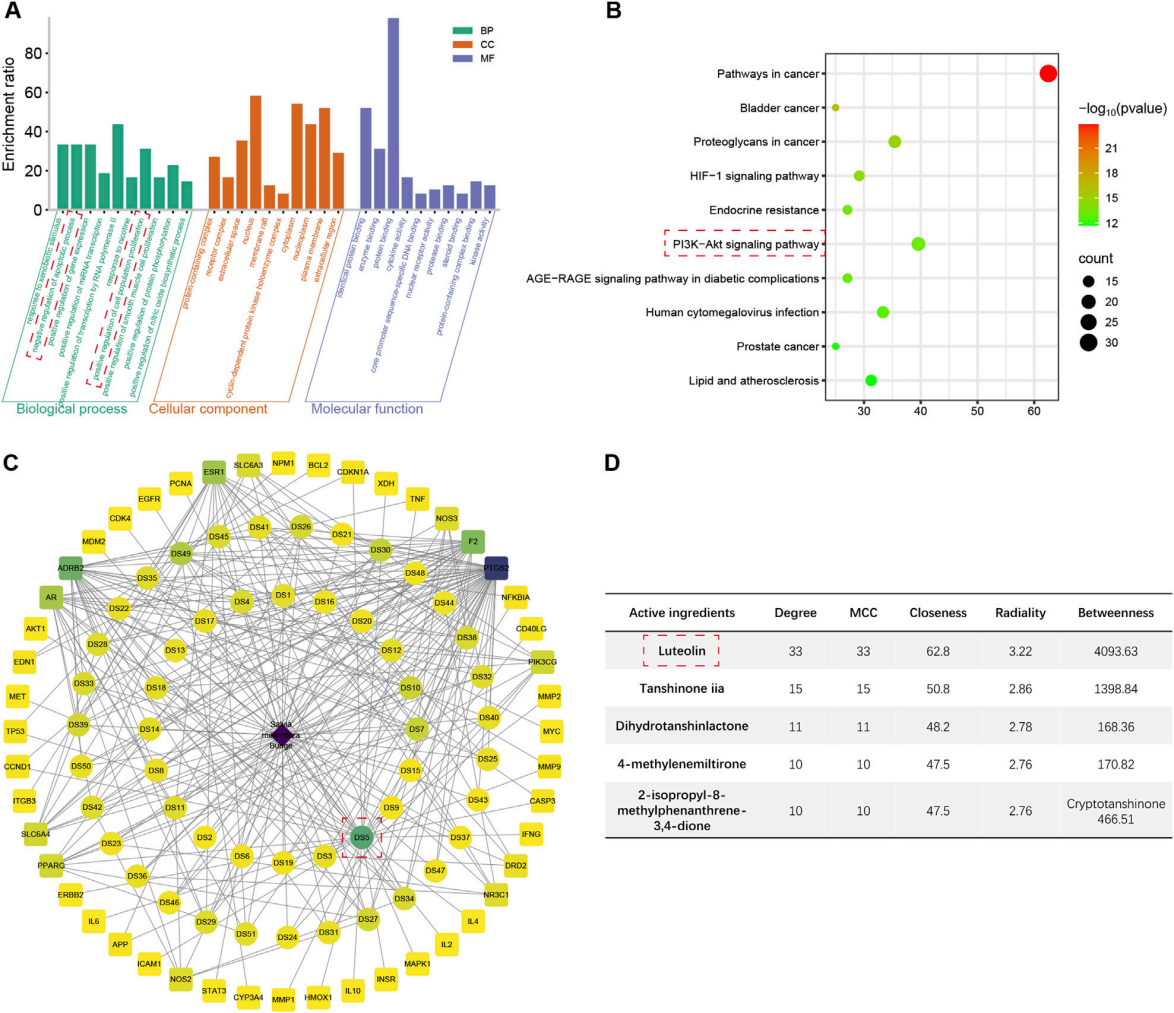
The results of network pharmacology analysis. **(A)** Top 10 enriched Go terms for genes shared between ALI/ARDS and *Salvia miltiorrhiza Bunge*. **(B)** Top 10 enriched KEGG pathways for genes shared between ALI/ARDS and *Salvia miltiorrhiza Bunge*. **(C)** Herb-active ingredient-shared gene network constructed by the shared genes between ALI/ARDS and *Salvia miltiorrhiza Bunge*. **(D)** Scoring of active ingredients using the cytoHubba plugin (2-isopropyl-8-methylphenanthrene-3,4-dione scores 142.02 in Betweenness).

The top 10 enriched terms and pathways from GO and KEGG enrichment analyses are presented in Figures 3A,B; Supplementary Figures S1B–G. The shared genes of the four herbs and ALI/ARDS were found to be associated with the regulation of gene expression, response to xenobiotic stimulus, regulation of apoptotic process, and regulation of cell population proliferation in GO terms. Regarding KEGG pathways, these genes were implicated in the AGE-RAGE signaling pathway in diabetic complications, pathways in cancer, lipid and atherosclerosis, and human cytomegalovirus infection. Specifically, the genes of ALI/ARDS shared with *S. miltiorrhiza Bunge* and *G. radix* were related to the negative regulation of the apoptotic process and the positive regulation of cell population proliferation, which were not observed in the genes of ALI/ARDS shared with *Radix Bupleuri* and *Scutellariae Radix*. Moreover, only the shared genes of ALI/ARDS and *S. miltiorrhiza Bunge* were associated with the HIF-1 pathway and the PI3K/Akt pathway. These pathways have been demonstrated to protect against LPS-induced ALI by mitigating inflammation and apoptosis (Shi et al., 2021; Huang CY. et al., 2020). Thus, *S. miltiorrhiza Bunge* emerged as an optimal candidate among the four herbs for screening hub active ingredients.

### 3.5 Hub active ingredient selection

The shared genes of ALI/ARDS and *S. miltiorrhiza Bunge* were employed to construct an herb-active ingredient-shared gene network (Figure 3C; Supplementary Table S8). The active ingredients were scored using the cytoHubba plugin (Figure 3D). Among the results obtained from different algorithms, the common active ingredients with high scores were identified as follows: luteolin, tanshinone iia, dihydrotanshinlactone, 4-methylenemiltirone, 2-isopropyl-8-methylphenanthrene-3,4-dione, and cryptotanshinone. Among the candidates, luteolin achieved the highest scores across various algorithms, thereby being identified as the hub active ingredient in the present study (Supplementary Table S9), which has the potential to exert a protective effect on ALI/ARDS by upregulating the expression of HGF.

### 3.6 Influence of luteolin on survival

**FIGURE 4:**
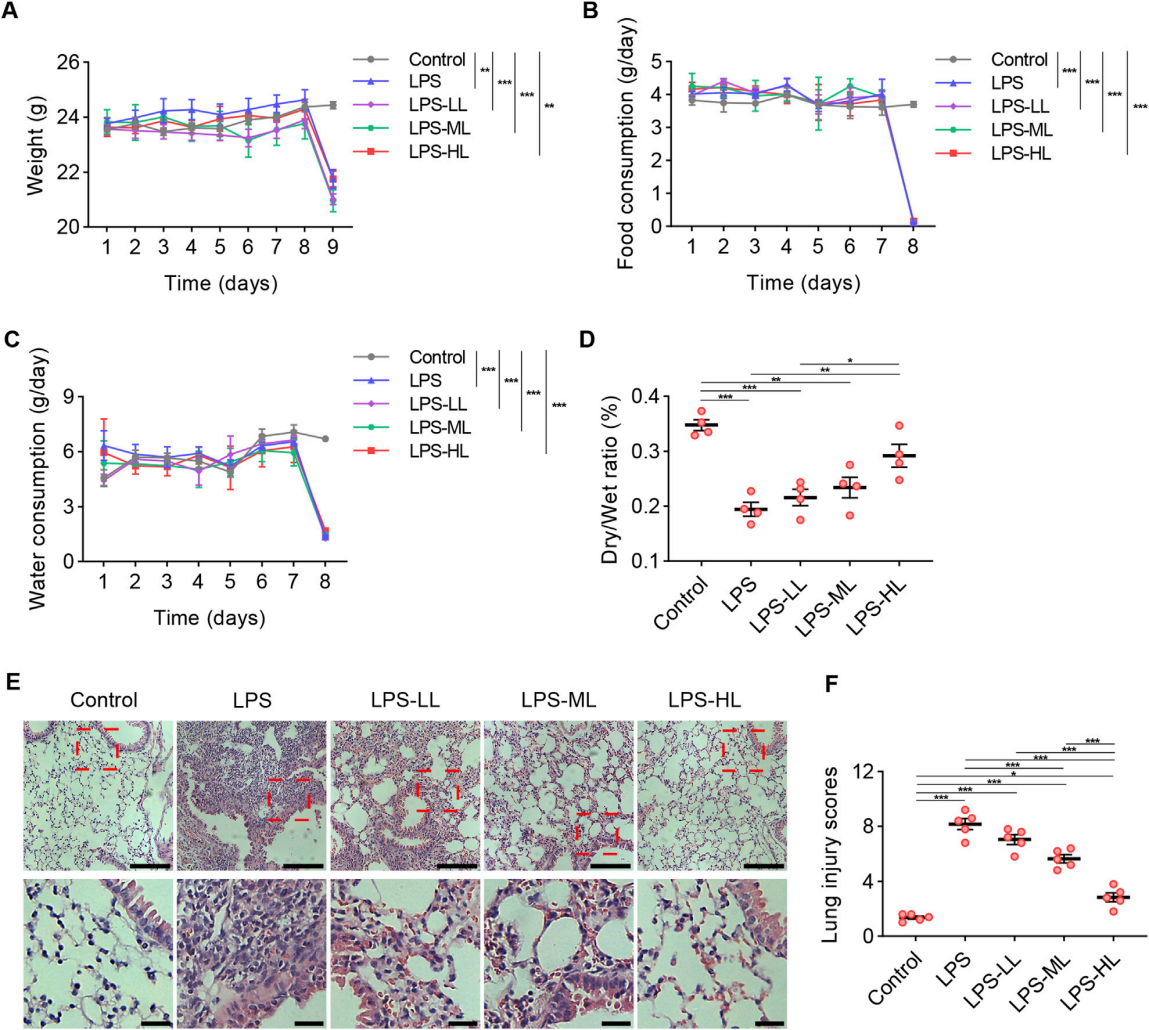
Luteolin alleviated pathological damages of lung in ALI mice. **(A)** Weight measurement over seven consecutive days. **(B)** Food intake measurement over seven consecutive days. **(C)** Water consumption measurement over seven consecutive days. **(D)** The lung dry/wet ratio. **(E)** Assessment of lung tissue pathological changes via H&E staining. **(F)** Lung injury scores based on H&E staining. 6 mice/group for A-C, 4 mice/group for D, 5 mice/group for F; Data were presented as means ± SD **(A–D,F)**; Repeated measures two-way ANOVA with Bonferroni post-hoc test **(A–C)**; One-way ANOVA with Bonferroni post-hoc test **(D,F)**; Scale bar: 100 μm, zoom-in pictures: 25 μm **(E)**; *P < 0.05, **P < 0.01, ***P < 0.001.

To evaluate the toxicity of luteolin at different dosages in mice, we measured the weight, food intake, and water consumption for seven consecutive days (Figures 4A–C). There were no significant differences in weight and the consumption of food or water between the mice treated with CMC-Na and those treated with luteolin. Due to the severe inflammatory responses induced by LPS administration, compared with the Control group, the mice that received LPS exhibited a significant reduction in weight, food intake, and water consumption. These results suggest that luteolin at different dosages has no toxic effects on mice over a 7-day period.

### 3.7 Luteolin alleviated pathological damages of lung in ALI mice

Luteolin treatment significantly increased the D/W ratio, suggesting that LPS-induced pulmonary edema and microvascular permeability were reduced by luteolin pretreatment (Figure 4D). H&E staining was conducted to assess the pathological changes in the lungs (Figures 4E,F). Compared with the mice that received saline, the lungs of ALI mice exhibited alveolar structure destruction, alveolar wall thickening, and inflammatory cell aggregation. In mice pretreated with luteolin, lung injury induced by LPS was significantly mitigated. These results indicate that luteolin exerts protective effects on alveolar-vascular barrier integrity, lung edema, and cellular damage in ALI. These protective effects were more pronounced in mice pretreated with high-dose luteolin.

### 3.8 Luteolin attenuated inflammation and apoptosis in ALI mice

**FIGURE 5:**
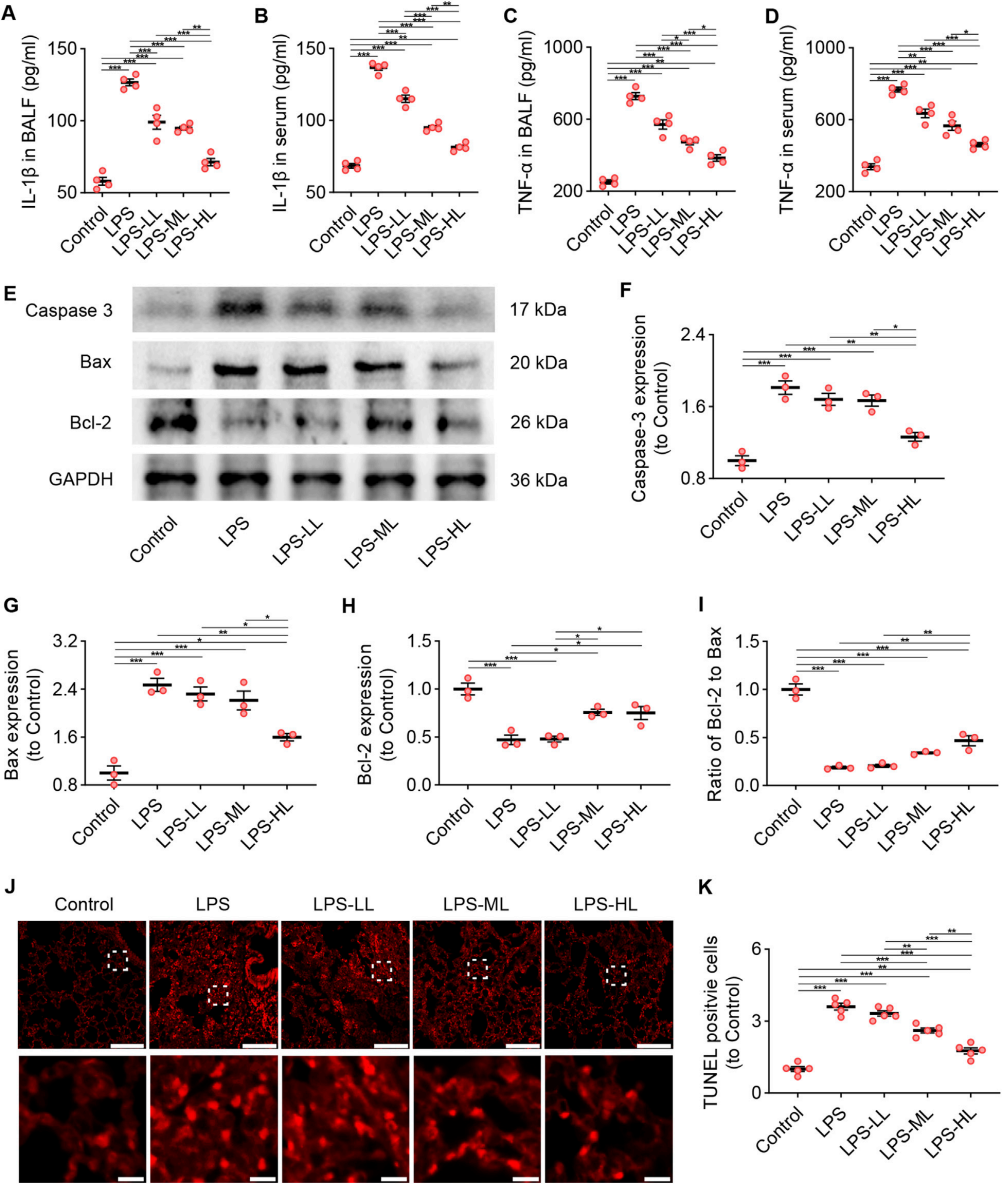
Luteolin attenuated inflammation and apoptosis in ALI mice. **(A)** The levels of IL-1β in BALF. **(B)** The levels of IL-1β in serum. **(C)** The levels of TNF-α in BALF. **(D)** The levels of TNF-α in serum. **(E)** The expression of Caspase 3, Bax, and Bcl-2 determined by Western blot. **(F)** Normalized expression of Caspase 3 based on Western blot. **(G)** Normalized expression of Bax based on Western blot. **(H)** Normalized expression of Bcl-2 based on Western blot. **(I)** The ratio of Bcl-2 to Bax based on Western blot. **(J)** Assessment of apoptosis in lung tissue using TUNEL staining. **(K)** Quantification of TUNEL-positive cells derived from TUNEL staining. 4 mice/group for **(A–D)**, 3 mice/group for **(F–I)**, 5 mice/group for **(K)**; Data were presented as means ± SD **(A–D,F–I,K)**; One-way ANOVA with Bonferroni post-hoc test **(A–D,F–I,K)**; Scale bar: 200 μm, zoom-in pictures: 20 μm **(J)**; *P < 0.05, **P < 0.01, ***P < 0.001.

The levels of the pro-inflammatory cytokines IL-1β and TNF-α were measured in BALF and serum using ELISA (Figures 5A–D). The results showed that the levels of IL-1β and TNF-α in BALF and serum of ALI mice were significantly higher than those in mice treated with saline. However, the pro-inflammatory responses were drastically reduced in ALI mice pretreated with luteolin. We also performed WB to determine the levels of Caspase-3, Bax, and Bcl-2 in the lungs to assess apoptosis (Figures 5E–I). Compared with the Control group, LPS-treated mice showed a significant increase in the expression of Caspase-3 and Bax, and a significant decrease in the expression of Bcl-2. However, luteolin pretreatment, especially with high dose, significantly enhanced the expression of Bcl-2 and reduced the expression of Caspase-3 and Bax. Consistent findings regarding apoptosis were also observed through TUNEL staining (Figures 5J,K). These results demonstrate that luteolin alleviates inflammation and apoptosis in the lungs of ALI mice.

### 3.9 Protection of luteolin on ALI was associated with PI3K/Akt pathway

**FIGURE 6:**
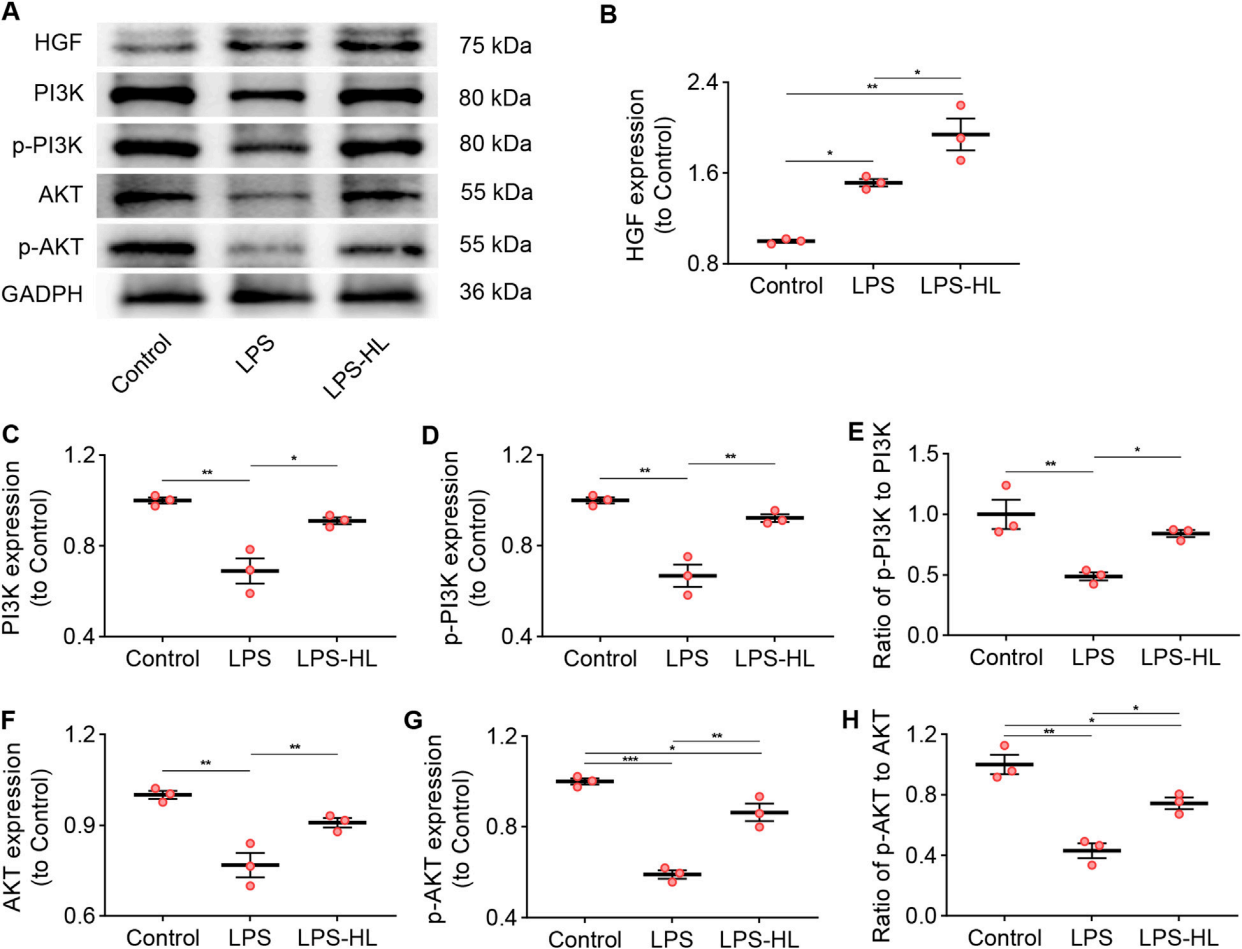
Protection of luteolin on ALI was associated with the PI3K/Akt pathway. **(A)** The expression of HGF, PI3K, p-PI3K, AKT, and p-AKT determined by Western blot. **(B)** Normalized expression of HGF based on Western blot. **(C)** Normalized expression of PI3K based on Western blot. **(D)** Normalized expression of p-PI3K based on Western blot. **(E)** The ratio of p-PI3K to PI3K based on Western blot. **(F)** Normalized expression of AKT based on Western blot. **(G)** Normalized expression of p-AKT based on Western blot. **(H)** The ratio of p-AKT to AKT based on Western blot. 3 mice/group for B-H; Data were presented as means ± SD **(B–H)**; One-way ANOVA with Bonferroni post-hoc test **(B–H)**; *P < 0.05, **P < 0.01, ***P < 0.001.

Compared with Control mice, the expression of HGF in the lungs was significantly increased in ALI mice treated with LPS, suggesting that there was an upregulation of HGF when the organism received insults (Zhu et al., 2018; Nakamura et al., 2000; Tang et al., 2020). We observed that the upregulated expression of HGF in the lung was stronger in ALI mice pretreated with luteolin, which may contribute to enhancing the antiapoptotic effects in ALI (Figures 6A,B).

Previous research has verified that HGF effectively suppresses apoptosis in both lung epithelial and endothelial cells by modulating the PI3K/Akt pathway (Panganiban and Day, 2011). Additionally, multiple studies have shown that activation of the PI3K/Akt pathway exerts protective effects against ALI through its anti-inflammatory and anti-apoptotic mechanisms (Panganiban and Day, 2011; Li et al., 2025; Zhang et al., 2022). Following KEGG pathway enrichment analysis, we identified that the shared genes of ALI/ARDS and *S. miltiorrhiza Bunge* were associated with the PI3K/Akt signaling pathway. WB results revealed that the expression of biomarkers for the PI3K/Akt pathway, including PI3K, p-PI3K, AKT, and p-AKT in the lungs of ALI mice were significantly lower than those in the Control group (Figures 6A,C–H). Conversely, the downregulated expression of these proteins was significantly increased in ALI mice pretreated with luteolin. These results suggest that activation of the PI3K/Akt signaling pathway is involved in the protective effects of luteolin on ALI.

### 3.10 Inhibition of c-Met attenuated the protective effects of luteolin on ALI

**FIGURE 7:**
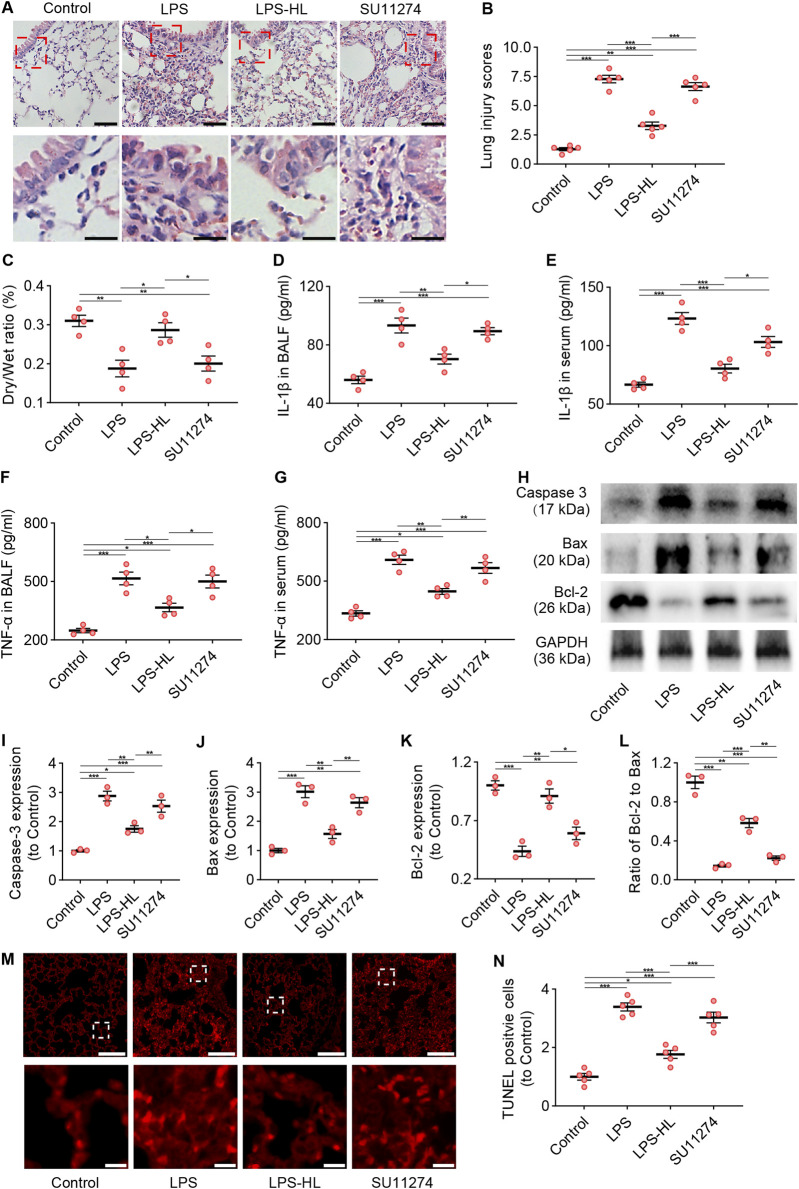
Inhibition of c-Met attenuated the protective effects of luteolin on ALI (SU11274 was administered intraperitoneally 30 min before high dose luteolin administration each day). **(A)** Assessment of lung tissue pathological changes via H&E staining. **(B)** Lung injury scores based on H&E staining. **(C)** The lung dry/wet ratio. **(D)** The levels of IL-1β in BALF. **(E)** The levels of IL-1β in serum. **(F)** The levels of TNF-α in BALF. **(G)** The levels of TNF-α in serum. **(H)** The expression of Caspase 3, Bax, and Bcl-2 determined by Western blot. **(I)** Normalized expression of Caspase 3 based on Western blot. **(J)** Normalized expression of Bax based on Western blot. **(K)** Normalized expression of Bcl-2 based on Western blot. **(L)** The ratio of Bcl-2 to Bax based on Western blot. **(M)** Assessment of apoptosis in lung tissue using TUNEL staining. **(N)** Quantification of TUNEL-positive cells derived from TUNEL staining. 5 mice/group for B and N, 4 mice/group for **(C–G)**, 3 mice/group for **(I–L)**; Data were presented as means ± SD **(B–G,I–L,N)**; One-way ANOVA with Bonferroni post-hoc test **(B–G,I–L,N)**; Scale bar: 100 μm, zoom-in pictures: 20 μm **(A)**; Scale bar: 200 μm, zoom-in pictures: 20 μm **(M)**; *P < 0.05, **P < 0.01, ***P < 0.001.

The tyrosine kinase receptor c-Met is a specific receptor for HGF, which is expressed on epithelial cells, endothelial cells, hepatocytes, and neurons (Tang et al., 2020; Mohamed et al., 2018; Chakraborty et al., 2013). HGF binds to c-Met, inducing c-Met dimerization and phosphorylation, which subsequently activates specific intracellular signaling cascades (Molnarfi et al., 2015; Mohamed et al., 2018). We inhibited c-Met using SU11274 to investigate the role of c-Met in the protective effects of luteolin on ALI. As shown in Figure 7, compared with ALI mice without luteolin treatment, mice pretreated with luteolin exhibited alleviated pathological damage and an increased D/W ratio in the lungs, reduced levels of IL-1β and TNF-α in BALF and serum, decreased expression of Caspase-3 and Bax, increased expression of Bcl-2, and reduced TUNEL-positive cells. However, the effects of luteolin on ALI in terms of improving pathological changes, anti-inflammation, and anti-apoptosis were abolished by c-Met inhibition. Taken together, luteolin attenuates inflammation and apoptosis in the lungs of ALI mice through the HGF/c-Met pathway.

## 4 Discussion

Although the pathogenesis of ALI/ARDS is complex, the inflammation and apoptosis play pivotal roles in the progression of this condition (Wang Y. et al., 2023; Han et al., 2025). In our study, by integrating MR analysis, network pharmacology analysis, and *in vivo* experiments, we observed that luteolin, an active ingredient extracted from *S. miltiorrhiza Bunge*, exhibits potential protective effects against LPS-induced ALI by upregulating the expression of HGF.

ALI/ARDS is a complex and critical illness characterized by decreased pulmonary compliance, increased alveolar capillary permeability, ventilation-perfusion imbalance, and pulmonary edema (Matthay et al., 2024; Bos and Ware, 2022). Bellani et al. reported that the incidence of ARDS among intensive care unit patients was 10.4% (Bellani et al., 2016). Another study indicated that the prevalence of mild and severe ARDS in medical/respiratory intensive care units was 9.7% and 47.4%, respectively (Huang X. et al., 2020). Despite significant progress in the treatment of ALI/ARDS, the mortality rate of patients with ALI/ARDS remains as high as 35%–46% (Bellani et al., 2016). Thus, there is an urgent need for innovative mechanisms and therapies to improve the outcomes of ALI/ARDS.

ALI/ARDS can be precipitated by a variety of factors, including infectious and non-infectious triggers, leading to direct lung injury due to local inflammation or indirect lung injury as a result of systemic inflammation (Bos and Ware, 2022; Qi et al., 2023). Previous studies have demonstrated that effective anti-inflammatory therapies are crucial for treating LPS-induced ALI in mice (Wang Y. et al., 2023; Han et al., 2025; Wang et al., 2022), providing therapeutic strategies to control the onset and progression of ALI. Consistent with previous studies (Wang Y. et al., 2023; Wang et al., 2022; Wang et al., 2024; Jia et al., 2023), we observed that ALI mice exhibited severe inflammatory responses during the development of ALI. We found that pretreatment with luteolin significantly downregulated the levels of IL-1β and TNF-α in both BALF and serum. Moreover, luteolin ameliorated the pathological damage of lung tissues in ALI mice. Luteolin has been demonstrated to exert anti-inflammatory effects in alveolar macrophages by inhibiting the activity of heat shock protein 90 and suppressing the production of pro-inflammatory cytokines (Chen C-Y. et al., 2007; Chen et al., 2014). Additionally, luteolin can activate regulatory T cells, thereby promoting the expression of IL-10, which in turn inhibits inflammation and mitigates lung injury induced by cecal ligation and puncture (Zhang et al., 2021). These findings suggest that luteolin provides protection against LPS-induced ALI through a series of synergistic actions *in vivo*, including the reduction of cytokine production and the preservation of the alveolar capillary barrier.

Excessive apoptosis of alveolar epithelial cells (AECs) plays a crucial role in the development of ALI/ARDS (Wang Y. et al., 2023; Wang et al., 2024; Yang et al., 2015; Hu et al., 2016). The apoptosis of AECs can induce damage to the alveolar epithelial barrier, resulting in alveolar cavity effusion and severe impairment of lung ventilation, ultimately promoting the progression of ALI/ARDS (Bem et al., 2007; Yang and Chen, 2023). Vitamin K2 can exert protective effects on ALI by upregulating Bcl-2 expression and downregulating Caspase-3 expression in LPS-induced ALI (Wang Y. et al., 2023). Inhibition of methyltransferase-like 3 can exert an anti-apoptotic effect, alleviating ALI by restoring neprilysin expression (Jia et al., 2023). These results suggest that approaches targeting anti-apoptotic regulation hold significant therapeutic potential for ALI/ARDS.

The treatment with MSCs significantly reduced the lung endothelial cell apoptosis by promoting the expression of HGF (Yang et al., 2015; Hu et al., 2016). In our study, using MR analysis, we found that higher HGF levels were associated with a reduced risk of ARDS. HGF is primarily secreted by mesenchymal cells and elicits proliferation, morphogenesis, and anti-apoptotic activity (Molnarfi et al., 2015; Watanabe et al., 2005; Ito et al., 2005). Numerous studies have demonstrated that HGF exerts therapeutic effects on diseases such as myocardial infarction, liver fibrosis, renal fibrosis, focal cerebral ischemia, and pulmonary fibrosis (Chaparro et al., 2015; Molnarfi et al., 2015; Ono et al., 2000; Wu et al., 2015; Feng et al., 2023). Through network pharmacology analysis, we found that luteolin was a hub active ingredient with a potential role in upregulating the expression of HGF. By conducting *in vivo* experiments, we observed that luteolin pretreatment upregulated the expression of HGF in damaged lung tissues of ALI mice.

HGF serves as an essential factor for tissue survival and regeneration by activating the c-Met receptor (Molnarfi et al., 2015; Watanabe et al., 2005). Activation of the HGF/c-Met pathway exerts an anti-apoptotic effect through promoting the expression of Bcl-xL in renal epithelial cells (Mesarosova et al., 2017). In the middle cerebral artery occlusion model, the reduction of infarction and neuronal apoptosis is achieved via activation of the HGF/c-Met/STAT3/Bcl-2 pathway (Tang et al., 2020). In our study, ALI mice pretreated with luteolin exhibited upregulated expression of the anti-apoptotic protein Bcl-2 and downregulated expression of the pro-apoptotic Bax and cleaved Caspase-3. Bcl-2 functions to inhibit the release of cytochrome c from mitochondria, and the cleavage of caspase-3 constitutes the key execution phase within the caspase cascade, thereby triggering apoptosis (Parrish et al., 2013). Another study also revealed that activation of the HGF/c-Met/mTOR pathway can partially ameliorate vascular endothelial damage in septic ALI animals (Peng et al., 2020). However, we found that the protective effects of luteolin on LPS-induced ALI, including alleviated pathological damage, increased lung D/W ratio, and reduced levels of IL-1β and TNF-α in BALF and serum, were all attenuated when c-Met was inhibited by SU11274. These results suggest that luteolin induces anti-inflammatory and anti-apoptotic effects in LPS-induced ALI mice through activation of the HGF/c-Met pathway.

Upon the binding of HGF to its high-affinity receptor c-Met, the kinase catalytic activity of the receptor is induced, which subsequently initiates a wide array of biological activities, including activation of the PI3K/Akt pathway (Molnarfi et al., 2015; Xiao et al., 2001). One study has confirmed that HGF inhibits apoptosis in lung epithelial and endothelial cells by regulating the PI3K/Akt pathway (Panganiban and Day, 2011). Another study has also revealed that the protective effect of remimazolam on ALI is attributed to its ability to suppress apoptosis through activation of the PI3K/Akt pathway (Li et al., 2025). Additionally, MSCs overexpressing glutamate cysteine ligase can ameliorate ALI, as activation of the PI3K/Akt pathway can reduce the expression of pro-apoptotic proteins and elevate the expression of anti-apoptotic proteins (Zhang et al., 2022). In the current study, the decreased expression of biomarker proteins associated with the PI3K/Akt pathway in the lungs of ALI mice was significantly upregulated when they were pretreated with luteolin. Taken together, activation of the HGF/c-Met/PI3K/Akt pathway plays an important role in the protective effects of luteolin on ALI.

## 5 Limitations

Although some novel insights into the protective effects of luteolin on LPS-induced ALI were obtained, several limitations should be considered in our study. First, although the MR results were validated by *in vivo* experiments, the estimates of the relationship between HGF and ALI/ARDS may not precisely correspond with the results obtained in clinical settings (Emdin et al., 2017). Second, we did not investigate the cellular origin of HGF in the lung, nor did we elucidate the underlying mechanism by which luteolin induces the expression of HGF in ALI mice. Third, network pharmacology analysis revealed that luteolin can target a variety of receptors or pathways. Thus, we should acknowledge that luteolin could exert protective effects on ALI by activating other receptors or pathways in synergy with the HGF/c-Met pathway. Fourth, we could not exclude the possibility that pathways other than the PI3K-Akt pathway indirectly impact the anti-apoptotic effect. Because the HGF/c-Met pathway is involved in many other mechanistic effects (Molnarfi et al., 2015; Panganiban and Day, 2011; Imamura and Matsumoto, 2017). Finally, the absence of experiments using PI3K/Akt pathway inhibitors and the lack of downstream effectors measurements limit the mechanistic exploration. Nevertheless, the anti-inflammatory and anti-apoptotic effects induced by luteolin in ALI mice are at least partially through activation of the HGF/c-Met/PI3K/Akt pathway.

## 6 Conclusion

In our study, we identified that elevated levels of HGF were associated with a decreased risk of ARDS using MR analysis. Luteolin was identified as a hub active ingredient associated with ALI/ARDS and HGF. Luteolin has potential protective effects on LPS-induced ALI through activation of the HGF/c-Met pathway. In the future, the downstream signaling pathways of the HGF/c-Met pathway should be further investigated to provide a theoretical basis for the prevention and treatment of ALI/ARDS using luteolin.

## Data Availability

The raw data supporting the conclusions of this article will be made available by the authors, without undue reservation.
