# Supplementary material for: Luteolin attenuates inflammation and apoptosis in LPS-induced ALI mice by activating the HGF/c-Met pathway
Source: Front Pharmacol. 2025 Jul 29;16:1641486. doi: 10.3389/fphar.2025.1641486 (PMC12339560; doi:10.3389/fphar.2025.1641486)
Supplement: Supplementary file 2 [file DataSheet1.docx]

**
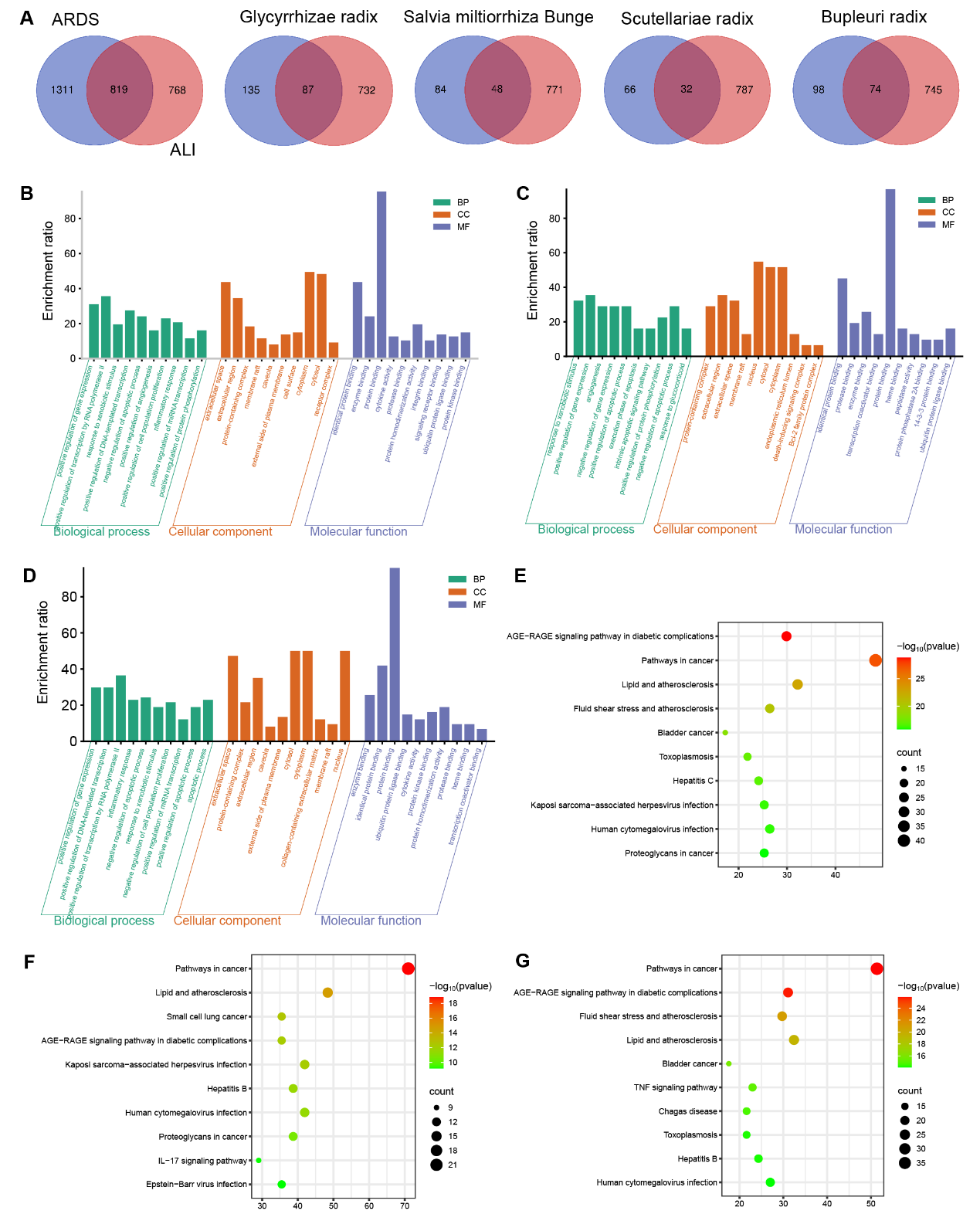
**

**Supplementary Figure 1** The results of network pharmacology analysis. (**A**) Identification of shared genes using Venn diagram analysis. (**B**) Top 10 enriched Go terms for genes shared between ALI/ARDS and *Glycyrrhizae radix*. (**C**) Top 10 enriched Go terms for genes shared between ALI/ARDS and *Scutellariae radix*. (**D**) Top 10 enriched Go terms for genes shared between ALI/ARDS and *Bupleuri radix*. (**E**) Top 10 enriched KEGG pathways for genes shared between ALI/ARDS and *Glycyrrhizae radix*. (**F**) Top 10 enriched KEGG pathways for genes shared between ALI/ARDS and *Scutellariae radix*. (**G**) Top 10 enriched KEGG pathways for genes shared between ALI/ARDS and *Bupleuri radix*.
